# Ultrasound characteristics of the mid-portion of the Achilles tendon in runners: a systematic review protocol

**DOI:** 10.1186/s13643-017-0501-z

**Published:** 2017-05-30

**Authors:** Prue Molyneux, Matthew Carroll, Sarah Stewart, Angela Brenton-Rule, Keith Rome

**Affiliations:** 1 Health and Rehabilitation Research Institute, Auckland University of Technology, 90 Akoranga Drive, Northcote 0627, Private Bag 92006, Auckland, 1142 New Zealand; 2 Faculty of Health and Environmental Sciences, School of Clinical Sciences, Department of Podiatry, Auckland University of Technology, 90 Akoranga Drive, Northcote 0627, Private Bag 92006, Auckland, 1142 New Zealand

**Keywords:** Achilles tendon, Ultrasound imaging, Running

## Abstract

**Background:**

Achilles tendinopathy is one of the most common overuse injuries in recreational and competitive runners, yet the clinical significance and frequency of abnormal sonographic characteristics in runners remains unclear. This paper presents a protocol for a systematic review which aims to assess existing literature which has employed ultrasonography to evaluate characteristics of the mid-portion of the Achilles tendon in runners.

**Methods:**

An electronic literature search will be conducted using the following electronic databases: MEDLINE, CINAHL and SPORTDiscus. Studies published in English will be included if they evaluate ultrasound characteristics associated with mid-portion Achilles tendinopathy in runners. Methodological quality will be assessed using a scale adapted from the Newcastle-Ottawa Scale.

**Discussion:**

This will be the first systematic review to summarise the existing evidence on ultrasound characteristics of the mid-portion of the Achilles tendon in recreational runners.

**Systematic review registration:**

PROSPERO CRD42016050509

**Electronic supplementary material:**

The online version of this article (doi:10.1186/s13643-017-0501-z) contains supplementary material, which is available to authorized users.

Achilles tendinopathy is a common musculoskeletal disorder characterised by pain and swelling in and around the Achilles tendon (AT) accompanied by impaired physical function [1]. AT injuries are the most common overuse syndrome of the lower limb, with a prevalence of up to 18% in recreational and competitive runners [2]. The condition has a cumulative lifetime incidence of approximately 24% in athletes. Running-related injuries have a prevalence between 11 and 85% or 2.5 to 59 per 1000 h of running [3]. The incidence of mid-portion Achilles tendinopathy in the general adult populations (21–60 years) has been reported with an incidence of 2.35 per 1000 and strongly associated with sporting activities [4]. The mid-portion of the AT, located 2 to 6 cm proximal to the calcaneal insertion, is the most commonly injured region, representing 55 to 65% of cases of Achilles tendinopathy [5]. It is likely this is due to the hypovascular nature of the tendon in this region which limits its ability to heal and regenerate after injury [6]. This is in contrast to insertional Achilles tendinopathy which is considered a different entity, with a different injury mechanism [7].

The AT is subject to considerable tensile and elastic loads during running which predominate in the mid-portion predisposing runners to Achilles tendinopathy [8]. The AT plays a crucial role in the transfer of calf muscle forces and production of coordinated movement. Alteration of the internal structure of the tendon by the degenerative process, which accompanies ageing, weakens the tissue when load exceeds its functional capacity, predisposing to further injury [9].

Musculoskeletal ultrasound imaging is a highly sensitive, reliable and non-invasive tool, and has been widely used to assess AT structure [10–12]. Ultrasonography may provide a clinical tool for the early detection of tendon abnormalities in the pre-symptomatic state, which may enable the identification of runners who have an increased risk of developing mid-portion Achilles tendinopathy. Currently, there is no standardised criteria that is used to stage tendinopathy according to intratendinous changes. Diagnostic ultrasound is regarded as the gold standard for imaging tendon-based injuries [13]. Several tendon characteristics can be assessed using ultrasound and include the presence of hypoechoic thickening, the loss of normal fibrillar pattern, irregularity of the tendon margins [14, 15], vascularisation [16], AT strain or displacement [17], and AT thickness and cross-sectional area (CSA) [18].

Sonographic tendon abnormalities are not always associated with patient-reported pain, with evidence suggesting that tendon degeneration starts long before the onset of symptoms [19, 20]. Up to 90% of tendons with ultrasound evidence of tendinopathic changes are asymptomatic [21, 22], and similar pathological features may be found in asymptomatic and symptomatic tendons [23]. Therefore, abnormal ultrasound findings may be present in the absence of clinical symptoms. Despite the important functional role of the AT in weight-bearing activity, particularly running, the clinical significance and frequency of abnormal sonographic characteristics in runners is unclear.

## Objective

The primary objective of this systematic review is to collect and appraise existing literature surrounding ultrasound characteristics of the mid-portion of the AT in runners and examine the effect that running volume has on the ultrasound characteristics. The secondary objective to appraise the scoring systems currently used to quantify ultrasound characteristics in the Achilles tendon.

## Methods

The review protocol is reported according to the Preferred Reporting Items for Systematic Review and Meta-Analysis Protocols (PRISMA-P) guidance, which can be found in (Additional file 1). The systematic review protocol was also registered with PROSPERO (CRD42016050509).

### Study design

The review will include observational studies, including case–control, longitudinal and cohort studies, which will facilitate the exploration of associations between ultrasound characteristics in runners. Unpublished, non-peer reviewed, intervention studies, studies that do not involve humans, in vitro studies, opinion articles, case series, letters to the editor, non-English articles, abstracts and case reports will be excluded.

### Eligibility criteria

Studies will be selected for inclusion in the systematic review according to the following criteria. Studies will be included with participants over 18 years of age who are classified as recreational runners. Participants will be described through ultrasound analysis as having mid-portion Achilles tendinopathy, Achilles tendinitis or tendinosis. Studies will be excluded if they involve participants with concomitant injury or pain developing from structures other than mid-portion of the AT (e.g. plantar heel pain, entheseal pathology). Studies which do not report ultrasound imaging localised to the mid-portion of the AT will be excluded. Studies will also be excluded if they include participants with a history of AT rupture or inflammatory arthritis or neurological, endocrine or metabolic disorders.

### Exposure

The exposure of interest will be running volume. If data is available, running characteristics, such as frequency, intensity and duration, will be reviewed.

### Outcomes

The outcomes of interest will be ultrasound characteristics of the mid-portion of the AT, directly measured by grey scale/B-mode, power Doppler, contrast-enhanced ultrasound (CEU) or elastography ultrasound imaging. The following outcomes will be assessed: cross sectional area (CSA), thickness, stiffness/strain, echoic change and vascularisation. The secondary outcome of interest will be the systems used to score the ultrasound characteristics of CSA, thickness, stiffness/strain, echoic change and vascularisation.

### Search strategy

A search will be conducted using the following electronic databases: MEDLINE, CINAHL and SPORTDiscus, using the search terms presented in Table 1. This search will be supplemented with hand-searching of reference lists of all potentially eligible full-text articles and selected review articles.

**Table 1:** Search strategy

a	1	Subject term	exp. Pathology
	2	Keywords	Achilles tend* or Achilles paraten* or Achilles mid-portion
b	3	Subject term	exp. Ultrasonography
	4	Keywords	Ultrasonograph* or Sonograph* or Ultrasound or US or MSUS or Doppler or Power Doppler or PDUS or Colour Doppler or Elastograph*
c	5	Subject term	exp. Running
	6	Keywords	Run* or Sprint* or Jog* or Interval or Long Distance or Marathon
d	7	Combine	1 or 2
	8	Combine	3 or 4
	9	Combine	5 or 6
	10	Combine	7 [and] 8 [and] 9

### Selection of studies

All titles and abstracts identified through database searches will be downloaded into EndNote version ×7 (Thomson Reuters, Philadelphia, PA, USA). The studies will be cross-referenced, and duplicates will be deleted. In the first stage of selection, all titles and abstracts of the studies will be screened for information fulfilling the inclusion and exclusion criteria by two independent reviewers (PM, MC). We will assess inter-rater agreement between investigators for study inclusion and methodological quality assessment using Kappa Cohen’s coefficient. The full-text content of all remaining articles will then be reviewed by the primary reviewer and by a second reviewer (MC). Any disagreement concerning the final inclusion/exclusion decision between the two authors will be resolved by consensus.

### Data extraction and management

The following information will be extracted from all included studies: study characteristics (study design, aim(s), outcome measure(s) reported and sample size) and participant characteristics (gender, mean age, mean body mass index). In addition, the mean distance run per week and years running will be recorded. Measurement techniques will be recorded including the ultrasound machine model, transducer size and imaging plane (transverse/longitudinal). Additionally, sonographic outcome measures related to the AT will be recorded including CSA (mm^2^), thickness (mm), stiffness/strain (kPa) and vascularisation (absent, mild, moderate, severe). The primary reviewer (PM) will extract these data and a second reviewer (MC) will independently check the extracted information. The data extraction form is detailed in (Additional file 2).

### Assessment of methodological quality

All studies will be independently assessed for methodological quality by the two reviewers using a scale adapted from the Newcastle-Ottawa Scale (NOS) [24]. The NOS contains eight items, categorised into three dimensions; selection, comparability and exposure (case–control studies) or outcome (cohort and cross-sectional studies). A star system is used to allow a semi-quantitative assessment of study quality. However, a maximum of two stars can be given for comparability. There are eight items to be identified in case–control and cohort studies. Therefore, case–control and cohort studies assigned 0–3, 4, 5–6 and 7–9 points will be identified as unsatisfactory, satisfactory, good and very good, respectively. There are five items used to assess cross-sectional studies, which will be assigned 0–2, 3, 4 or 5 points to identify them as unsatisfactory, satisfactory, good or very good, respectively. An additional file shows the NOS in detail (see Additional file 3). The final quality score for each study will be resolved by consensus from two reviewers.

### Statistical analysis

Given the anticipated paucity of literature surrounding this topic, the review is intended to be descriptive in nature. To address the differentiation of ultrasound characteristics and running volume, where appropriate and if studies are homogeneous, meta-analyses will be conducted by pooling mean differences and odds ratio (OR) using a random-effects model. If data from the study is not appropriate for meta-analyses, a narrative presentation or summary will be included. With regard to the second objective of the systematic review surrounding analysis of the ultrasound scoring systems used to quantify ultrasound characteristics, a narrative summary of results will be undertaken.

## Discussion

The increase in running volume inherent in long-distance runners predisposes them to overuse injuries, with the AT being the most commonly effected in the lower limb. There is a lack of understanding concerning which ultrasound characteristics may develop as an adaption to increased running volume or whether they indicate a susceptibility to clinical manifestations of Achilles tendinopathy. In addition, results from this review may direct the development of future prospective studies which examine the relationship between symptomatic Achilles tendinopathy and the presence of morphological and mechanical alterations of the AT.

### Limitations

The literature that is likely to be identified by the search strategy is likely to include publications with small sample sizes, low methodological quality, poorly defined categorisation of running, non-standardisation of scoring systems and variation in imaging machinery and imaging protocols. Consequently, we have included a broad definition of types of running, limited the investigation to the mid-portion of the AT and assessed a wide range of pathologies that can manifest in the mid-portion of the AT.

## Additional files

Additional file 1: PRISMA-P checklist

Additional file 2: Data extraction form

Additional file 3: Methodological quality assessment tool—Newcastle-Ottawa Scale

## Abbreviations

AT: Achilles tendon
CEU: Contrast-enhanced ultrasound
CSA: Cross-sectional area
NOS: Newcastle-Ottawa Scale
