# Supplementary material for: Ultrasound characteristics of the mid-portion of the Achilles tendon in runners: a systematic review protocol
Source: Syst Rev. 2017 May 30;6:108. doi: 10.1186/s13643-017-0501-z (PMC5450404; doi:10.1186/s13643-017-0501-z)
Supplement: Supplementary file 2 — Data extraction form [file 13643_2017_501_MOESM2_ESM.pdf]

Additional File 2: Data extraction form

Study title			
Study design			
Study aim			
Sample size (n)	M		
	F		
Participant characteristics	Age	M	
		F	
	Height	M	
		F	
	Weight	M	
		F	
	BMI	M	
		F	
Ultrasound mode			
Ultrasound characteristic reported			
Ultrasound scoring system	Quantitative		
	Qualitative		
Ultrasound acquisition	Machine model		
	Transducer		
	Imaging plane		
Ultrasound outcome measures	Cross-sectional area		
	Thickness		
	Vascularisation		
	Stiffness		
Running type	Volume		
	Frequency		
	Intensity		
	Duration		
